# Popular Ignorance of Medicine

**Published:** 1870-02

**Authors:** 


Popular Ignorance of Medicine.—A recent number of the New York *Nation* contains this pregnant sentence: "The greatest difficulties in the physician's practice arise from the dense ignorance of even the best classes of society as to the nature and cause of disease and the effects of misconduct, and it is deeply to be regretted that medical reading does not form some part of every person's self-culture or education."

Who is to blame that it does not form some part? None so much as physicians themselves. There is on the part of a number of medical men a jealousy about imparting information on medicine. They fear it may hurt their practice, perhaps, but they *say* the public may be injured by it. The success of quacks and charlatans is in direct proportion to the ignorance of a community on medical matters, and they always succeed best in those branches of medicine where sound instruction is most carefully withheld. Let physicians learn, that truth is no more to be feared in physiology than in religion.

